# *Withania somnifera* Induces Cytotoxic and Cytostatic Effects on Human T Leukemia Cells

**DOI:** 10.3390/toxins8050147

**Published:** 2016-05-12

**Authors:** Eleonora Turrini, Cinzia Calcabrini, Piero Sestili, Elena Catanzaro, Elena de Gianni, Anna Rita Diaz, Patrizia Hrelia, Massimo Tacchini, Alessandra Guerrini, Barbara Canonico, Stefano Papa, Giovanni Valdrè, Carmela Fimognari

**Affiliations:** 1 Department for Life Quality Studies, Alma Mater Studiorum-University of Bologna, Corso d’Augusto 237, 47921 Rimini, Italy; eleonora.turrini@unibo.it (E.T.); cinzia.calcabrini@unibo.it (C.C.); elena.catanzaro2@unibo.it (E.C.); elena.degianni2@unibo.it (E.d.G.); 2 Department of Biomolecular Sciences, University of Urbino Carlo Bo, Via Saffi 2, 61029 Urbino, Italy; piero.sestili@uniurb.it (P.S.); anna.diaz@uniurb.it (A.R.D.); barbara.canonico@uniurb.it (B.C.); stefano.papa@uniurb.it (S.P.); 3 Department of Pharmacy and Biotechnology, Alma Mater Studiorum-University of Bologna, Via Irnerio 48, 40126 Bologna, Italy; patrizia.hrelia@unibo.it; 4 Department of Life Sciences and Biotechnology (SVeB)-LT Terra & Acqua Tech RU, University of Ferrara, Corso Ercole I d’Este 32, I-44121 Ferrara, Italy; massimo.tacchini@unife.it (M.T.); grrlsn@unife.it (A.G.); 5 Department of Biological, Geological and Environmental Sciences (BiGeA), Alma Mater Studiorum-University of Bologna, Piazza di Porta S. Donato 1, 40126 Bologna, Italy; giovanni.valdre@unibo.it

**Keywords:** *Withania somnifera*, apoptosis, cell cycle, leukemia, oxidative stress, immunogenic cell death, genotoxicity

## Abstract

Cancer chemotherapy is characterized by an elevated intrinsic toxicity and the development of drug resistance. Thus, there is a compelling need for new intervention strategies with an improved therapeutic profile. Immunogenic cell death (ICD) represents an innovative anticancer strategy where dying cancer cells release damage-associated molecular patterns promoting tumor-specific immune responses. The roots of *Withania somnifera* (*W. somnifera*) are used in the Indian traditional medicine for their anti-inflammatory, immunomodulating, neuroprotective, and anticancer activities. The present study is designed to explore the antileukemic activity of the dimethyl sulfoxide extract obtained from the roots of *W. somnifera* (WE). We studied its cytostatic and cytotoxic activity, its ability to induce ICD, and its genotoxic potential on a human T-lymphoblastoid cell line by using different flow cytometric assays. Our results show that WE has a significant cytotoxic and cytostatic potential, and induces ICD. Its proapoptotic mechanism involves intracellular Ca^2+^ accumulation and the generation of reactive oxygen species. In our experimental conditions, the extract possesses a genotoxic potential. Since the use of *Withania* is suggested in different contexts including anti-infertility and osteoarthritis care, its genotoxicity should be carefully considered for an accurate assessment of its risk–benefit profile.

## 1. Introduction

Cancer causes millions of deaths every year. Only in 2012, cancer deaths have reached 8.2 million. In 2030, 12.6 million cancer deaths have been estimated [1].

Cancer originates from multiple alterations induced by a direct interaction between toxic agents and DNA. This triggers gene and chromosome mutations. Altered expressions of oncogenes and tumor suppressor genes are found in different cancer types. The consequence is an uncontrollable proliferation mediated by growth signals released from the tumor cells themselves, resistance against antigrowth signals, and inhibition of apoptosis. Furthermore, cancer expansion is helped by the release of various growth factors which lead to the formation of new blood vessels that provide nutrients and oxygen thus favoring cancer spread and metastasis dissemination [2]. Despite the progress made in anticancer research, traditional cytotoxic chemotherapy continues to serve as the basis for the current standard therapeutic regimen. This is characterized by an elevated intrinsic toxicity, mainly due to its poor selectivity for cancer cells. Furthermore, cancer cells’ ability to develop drug resistance represents a major problem in anticancer therapy [3]. Thus, there is a compelling need for new intervention strategies with an improved therapeutic profile. Cancer cells create a favorable microenvironment allowing them to survive, proliferate, and counteract immunosurveillance. A promising anticancer strategy could be represented by the use of cytotoxic drugs that are not only able to induce tumor cell death, but also promote tumor-specific immune responses, potentially preventing tumor progression and relapse [4]. Very recent studies have introduced the concept of immunogenic cell death (ICD), a modality of cell death where, after the exposure to some cytotoxic agents, dying cancer cells release endogenous damage-associated molecular patterns (DAMPs) including calreticulin, heat shock protein (Hsp)-70 and Hsp-90 recognized by antigen-presenting cells such as dendritic cells (DCs). This is followed by T-cell-mediated adaptive immunity [5].

Natural products represent a rich source of biologically active compounds that can be able to interact simultaneously with different targets involved in cell growth, cell differentiation, and apoptosis regulation [6]. In 2015, the Food and Drug Administration (FDA) released an updated guidance on botanical drug development. Unlike drugs that are constituted by a single active ingredient, botanical drugs have a heterogeneous nature, which may lead to uncertainty in relation to their active constituents. The number of botanical products submitted to the FDA is particularly high in the oncological area [7]. The first botanical drug in this area is Polyphenon E, a standardized extract obtained from the leaves of green tea (*Camellia sinensis*), approved by the FDA in 2007 for treatment of genital warts linked to human papilloma viruses. The well-defined make-up, standardization, and cheap cost make Polyphenon E a very interesting candidate for human clinical studies. Polyphenon E is currently in several trials as a chemopreventive and chemotherapeutic agent against chronic lymphocytic leukemia, bladder and lung cancers (phase II), and in breast cancer (phase I) [8,9,10].

Many recent studies focus on the potential anticancer effect of crude extracts from plants used in traditional medicine and their isolated compounds. The roots of *Withania somnifera* (*W. somnifera*), a plant originating from Asia and South Africa [11], are used in the Indian traditional medicine [12]. A wide range of biological activities is reported for *W. somnifera* including anti-inflammatory [13], immunomodulating [14], neuroprotective [15], and anticancer activities [16]. The present study is designed to explore the antileukemic activity of the DMSO extract obtained from the roots of *W. somnifera* (WE). Particular emphasis is given to the role of reactive oxygen species (ROS) in its anticancer effect. With the aim to extend the potential clinical impact of *Withania*, we investigated its ability to induce ICD and assessed on a preliminary basis the risk–benefit profile associated with the use of this plant through the analysis of its genotoxic potential.

## 2. Results

### 2.1. WE Contains Withaferin A (WFA), Whitanolide A (WDA), Withanolide B in Trace Amount

We detected and quantified WFA and WDA (Table 1), which are among the most representative markers of *Withania somnifera* [17]. WFA was also described as highly soluble in DMSO, confirming our results. Withanolide B was instead under the Limit Of Quantification (LOQ = 4.36 ± 0.65 μg/mL) and withanone undetectable.

### 2.2. WE Induces Apoptosis and Alters Cell-Cycle Residence

WE causes a dose-dependent reduction of cell viability. For example, after 24 h treatment of Jurkat cells with 1.6 mg/mL of WE, the percentage of viable cells was 64.4% and at 3.2 mg/mL cells viability achieved 16.6%. The calculated IC_50_ value (the inhibitory concentration causing cell toxicity by 50% following one cell-cycle exposure) was 2.3 mg/mL. Concentrations similar or smaller than the IC_50_ were used in the following experiments.

**Figure 1:**
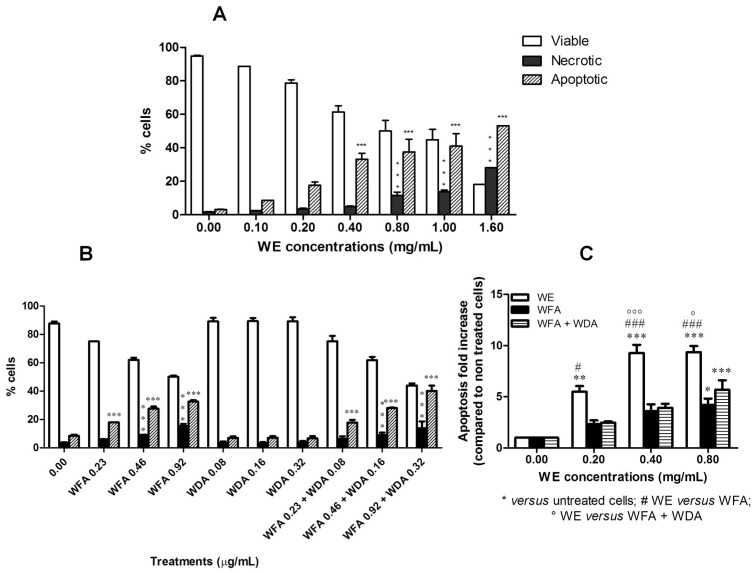
Percentage of viable, necrotic and apoptotic cells after 24 h treatment of Jurkat cells with increasing concentrations of: DMSO extract obtained from the roots of *W. somnifera* (WE) (**A**); and withaferin A (WFA), withanolide A (WDA) or WFA plus WDA (**B**). Fold increase in the percent of apoptotic cells after treatment with different concentrations of WE, WFA, or WFA plus WDA (**C**). * *p* < 0.05; ** *p* < 0.01; *** *p* < 0.001 *versus* untreated cells.

Further analyses were carried out to discriminate whether the inhibitory effect of WE on cell viability was the result of apoptotic cell death. After 6 h of treatment at 0.4 and 0.8 mg/mL, WE significantly increased the percent of apoptotic cells (3.4- and 4.1-fold increase, respectively, *versus* untreated cells). After 24 h of treatment, the percent of apoptotic cells was statistically significant starting from 0.4 mg/mL, where 33.1% ± 3.7% of apoptotic events was observed *versus* 3.1% ± 0.2% of untreated cells (Figure 1). An increase in necrotic events was also recorded starting from 0.80 mg/mL (11.6% ± 1.9% *versus* 1.7% ± 0.2% of untreated cells). At the highest tested concentration of WE (1.6 mg/mL), both apoptotic and necrotic events markedly increased, but the percentage of apoptotic cells was significantly higher than that of necrotic cells (53.2% *versus* 28.2%, respectively) (Figure 1A). When cells were treated with WFA, WDA or their association, we observed an increase in the fraction of apoptotic cells only for WFA at all the concentrations tested (Figure 1B). The proapoptotic effect of the association WFA plus WDA was very similar to that of WFA (Figure 1B). In Figure 1C, we compared the fold increase in the percent of apoptotic cells recorded after treatment with WE, WFA or WFA plus WDA. The concentrations of WFA and WDA are those found in the extract at 0.20, 0.40 and 0.80 mg/mL. Even if WFA and WFA plus WDA possess a proapoptotic effect, the effect of WE is significantly higher than that observed for WFA or the association.

**Figure 2:**
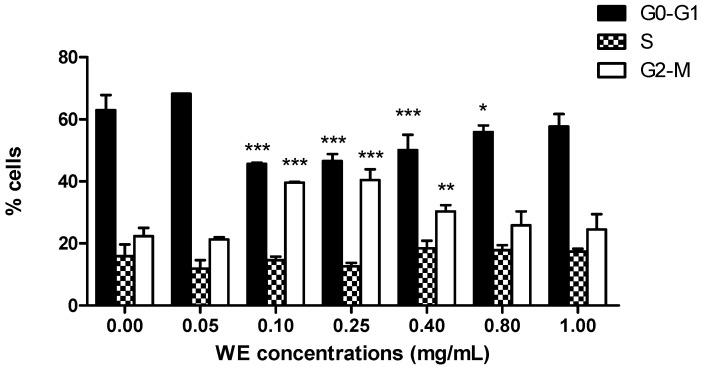
Cell-cycle distribution following 24 h treatment of Jurkat with increasing concentrations of WE. * *p* < 0.05; ** *p* < 0.01; *** *p* < 0.001 *versus* untreated cells.

In the following experiments, we highlighted the cytostatic effect of WE. After 24 h treatment at increasing concentrations of WE, we observed an increasing number of cells in G2/M phase starting from 0.1 mg/mL (39.6% ± 0.1% *versus* 22.4% ± 1.2% of untreated cells), accompanied by a decrease in cells in phase G0/G1 (45.7% ± 0.1% *versus* 63.0% ± 2.2% of untreated cells) (Figure 2). WE showed the same trend up to 0.4 mg/mL, where we detected an increase in cells in G2/M phase (30.3% ± 0.9%) and a decrease in cells in G0/G1 phase (50.1% ± 2.2%). At the highest concentrations tested, the cell-cycle distribution was similar to that of untreated cells (Figure 2).

### 2.3. WE Increases Intracellular Ca^2+^ ([Ca^2+^]_i_)

**Figure 3:**
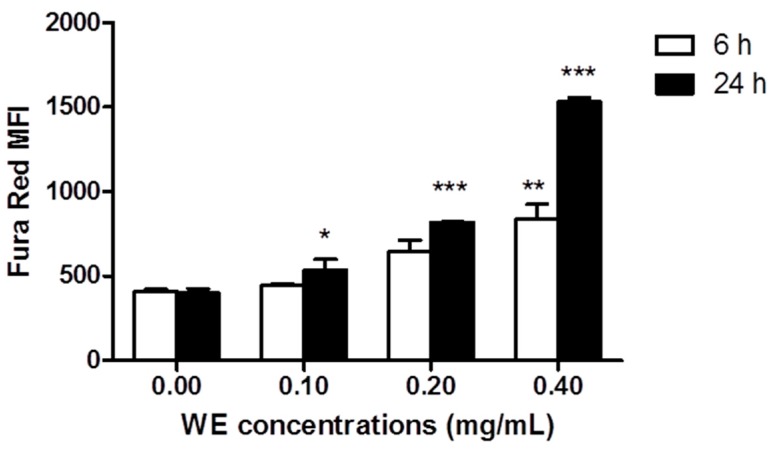
Fraction of living cells with increased [Ca^2+^]*_i_* following 6 and 24 h exposure to increasing concentrations of WE. * *p* < 0.05; ** *p* < 0.01; *** *p* < 0.001 *versus* untreated cells.

We explored the ability of WE to modulate [Ca^2+^]*_i_* on viable cells after 6 and 24 h of Jurkat treatment with WE. The extract increased [Ca^2+^]*_i_* in a dose- and time-dependent manner. At 6 h, [Ca^2+^]*_i_* was significantly enhanced only at the highest concentration (0.4 mg/mL) tested [837.5 ± 86.9 MFI (mean fluorescence intensity) *versus* 410.5 ± 12.4 MFI of untreated cells] (Figure 3). After 24 h of WE, we recorded a significant increase in [Ca^2+^]*_i_* at all tested concentrations, starting from 0.1 mg/mL (535.5 ± 61.5 MFI *versus* 399.7 ± 26.9 MFI of untreated cells) and becoming 3.7 fold higher than control at the highest tested concentration (1530 ± 27.7 MFI). Dead cells were analyzed separately as unique cluster. We observed an increase in [Ca^2+^]*_i_* (data not shown) that confirms the involvement of Ca^2+^ in the antileukemic effect of WE.

### 2.4. WE Induces Oxidative Stress

**Figure 4:**
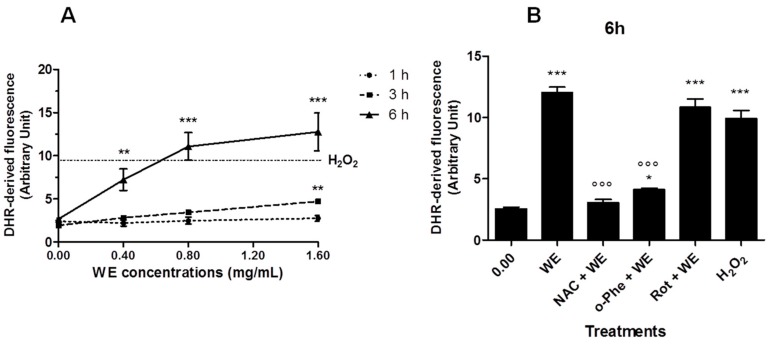
Reactive oxygen species (ROS) generation in WE-treated cells: (**A**) Jurkat exposed to increasing concentrations of WE for 1 h, 3 h or 6 h. Cells treated with H_2_O_2_ 0.1 mM for 15 min represent the positive control (dotted line parallel to *x*-axis). (**B**) Cells were treated for 6 h with WE 0.8 mg/mL in the absence or presence of o-phenanthroline (o-Phe, 10 μM), rotenone (Rot, 2 μM) or *N*-acetylcysteine (NAC, 10 mM). Cells treated with H_2_O_2_ 0.1 mM for 15 min represent the positive control. * *p* < 0.05, ** *p* < 0.01, *** *p* < 0.001 versus control, and °°° *p* < 0.001 versus WE.

WE extract increased ROS production in a dose-dependent manner in Jurkat cells (Figure 4A). Most of the ROS were generated between 3 and 6 h of incubation. After 6 h treatment with WE, 0.8 and 1.6 mg/mL of WE led to ROS levels similar or even higher than those promoted by a mildly toxic dose of H_2_O_2_ (0.1 mM for 15 min), included as a positive control. After longer times of treatment (18 and 24 h), ROS generation reached a plateau (data not shown). *N*-acetylcysteine (NAC) and o-phenantroline (o-Phe) significantly inhibited the ROS generation induced by WE (0.8 mg/mL for 6 h), while rotenone (Rot) was unable to afford a protective effect (Figure 4B).

### 2.5. Co-Treatment of Cells with WE and NAC Significantly Decreases WE-Induced Apoptosis

**Figure 5:**
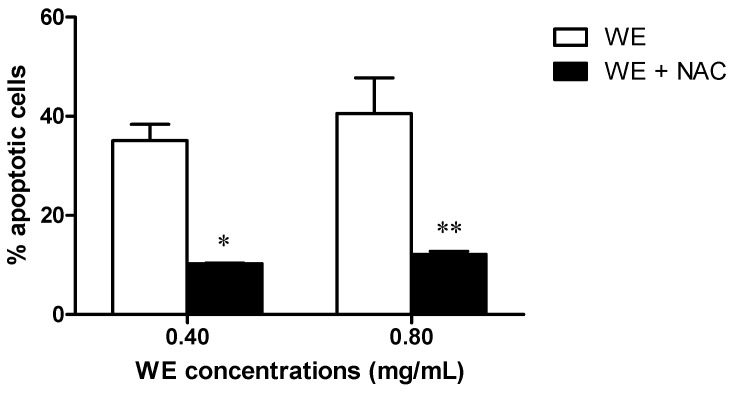
Apoptotic events after 24 h of Jurkat treatment with WE in the absence and presence of *N*-acetyl cysteine (NAC) (10 mM). * *p* < 0.05; ** *p* < 0.01 *versus* WE.

Because of the crucial role of ROS in the bioactivity of WE, we investigated whether the alteration of the redox state induced by NAC treatment could play a role in the apoptosis induced by WE. We observed a significant decrease in the WE-induced apoptotic events following 24 h of Jurkat co-treatment with WE plus NAC (10 mM). The WE-induced apoptotic events were significantly reduced from 35% after 0.40 mg/mL of WE to 10% after WE plus NAC and from 38% after 0.80 mg/mL of WE to 12% after WE plus NAC (Figure 5).

Moreover, we co-treated cells with WE and l-asparagine (1–2 mM): under this condition, we did not record any modulation of the proapoptotic potential of WE (data not shown).

### 2.6. WE Induces ICD

**Figure 6:**
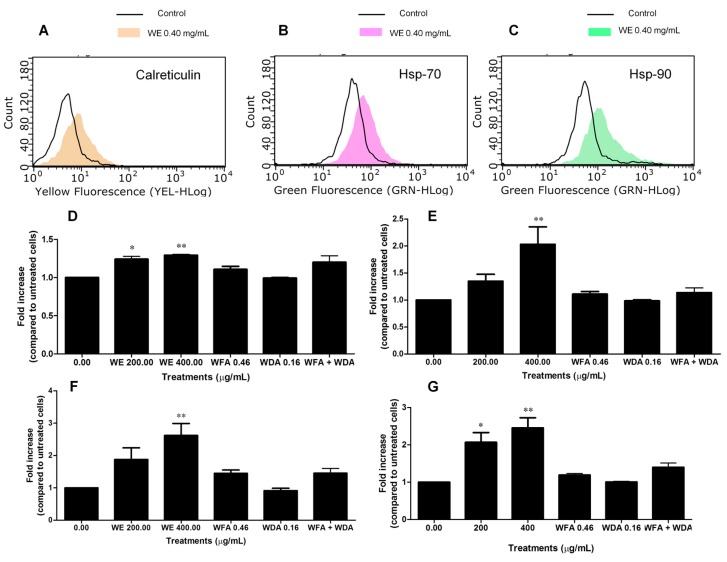
Fluorescence hystograms of: immunolabeled calreticulin (**A**); Hsp-70 (**B**); and Hsp-90 (**C**). Modulation of the expression of: calreticulin (**D**); Hsp-70 (**E**); Hsp-90 (**F**); and of ATP release (**G**) after treatment with WE, WFA, WDA or WFA plus WDA. Histograms are representatives of three independent experiments. * *p* < 0.05, ** *p* < 0.01 *versus* untreated cells.

Based on the cytotoxic activity of WE and its ability to increase intracellular ROS and Ca^2+^ levels, we preliminarily explored the capacity of WE to induce ICD. To this aim, the exposure of some DAMPs on the extracellular membrane of Jurkat cells was examined. After 6 h of treatment, we did not observe any effect of WE on calreticulin translocation (data not shown). Longer treatment times (24 h) caused calreticulin translocation on the extracellular membrane (Figure 6A,D), with a mean fluorescence of 8.39 at 0.2 mg/mL and 8.72 at 0.4 mg/mL compared to 6.83 of the control (Figure 6A). Similarly, cells treated with WE showed an increase in both Hsp-70 and Hsp-90 expression only after 24 h of treatment (Figure 6E,F, respectively). As an example, at 0.2 mg/mL Hsp-70 fluorescence was 57.92 compared to 42.17 of the control and Hsp-90 mean fluorescence was 144.14 compared to 58.45 of untreated cells; at 0.4 mg/mL, the fluorescence of both Hsp-70 and Hsp-90 increased to 85.22 and 151.09, respectively (Figure 6B,C, respectively). Finally, we measured the release of adenosine triphosphate (ATP) from dying cells after 6 and 24 h of treatment with WE. At 6 h, we did not record any modulation of ATP levels (data not shown). After 24 h, we observed a significant increase in ATP levels at both the tested concentrations of the extract (2- and 2.45-fold increase, respectively) (Figure 6G).

We also tested the induction of ICD by the two main constituents of WE extract (*i.e.*, WFA and WDA), used at the concentrations found in the WE extract at 0.40 mg/mL. Treatment with WFA and WDA alone or in association for 24 h did not cause a statistically significant modulation of calreticulin translocation, Hsp-70 and Hsp-90 expression or ATP release (Figure 6D–G).

### 2.7. WE Induces DNA Damage

**Figure 7:**
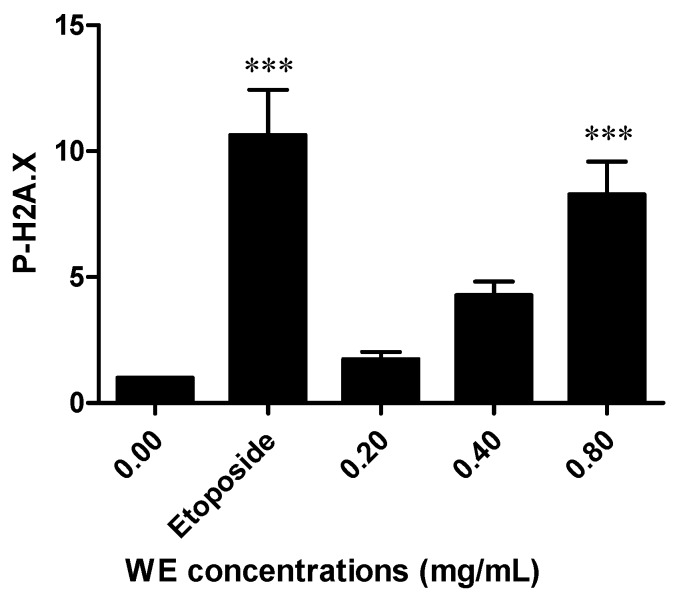
Relative expression of phosphorylated H2A.X (P-H2A.X) induced by WE in Jurkat cells after 6 h of treatment. Etoposide (10 μM) was used as positive control. *** *p* < 0.001 *versus* untreated cells.

To evaluate the ability of WE to induce DNA damage, H2A.X phosphorylation was analyzed. H2A.X phosphorylation at Ser 139 represents a sensitive marker for DNA strand breakage [18]. WE induced a dose-dependent increase in H2A.X phosphorylation, which was eight times higher than untreated cells at the highest tested concentration (0.80 mg/mL). This increase was similar to the phosphorylation induced by etoposide 10 μM, used as positive control (Figure 7).

## 3. Discussion

In this study, we demonstrated the *in vitro* antileukemic effect of the root extract of *W. somnifera* in a T-lymphoblastoid cell line. The high-performance liquid chromatography (HPLC) analysis performed on WE revealed the presence of WFA, WDA and to a lesser extent withanolide B. Withanolides, in particular WFA and its acetyl derivative, are highly bioactive and show anticancer activity [19,20,21]. Of note, its dihydroderivative is not active, thus suggesting that an unsaturated lactone moiety in ring A of WFA is important for its biological activity. In our experimental settings, WE significantly induced apoptosis in a remarkable proportion of cells. Moreover, it blocked cell proliferation through an accumulation of cells in the G2/M phase starting from the lowest tested concentrations. Cell-cycle dysregulation represents a hallmark of cancer [22] and targeting the checkpoint signaling pathway, which usually leads to an arrest at G1/S or G2/M boundaries, is an effective therapeutic strategy [23]. Our results confirm the antiproliferative and proapoptotic effect reported for withanolides and for a methanolic crude *Withania* leaf extract in different leukemia cell lines [24,25]. However, the IC_50_ calculated for the above mentioned methanolic leaf extract was much lower than that calculated in our study. The difference could be imputable to the different part of plant (root *versus* leaf) used and/or the method of extraction (the methanolic leaf extract was subjected to a sequential solvent extraction, which progressively concentrated the active components of *Withania* leaves).

Different mechanisms can be involved in the proapoptotic activity of our WE. Numerous studies reported that intracellular Ca^2+^ mobilization plays a crucial role in apoptosis [26,27] and that calcium ionophores exhibit proapoptotic activity [28]. Since WE exhibited a marked proapoptotic ability, we measured [Ca^2+^]*_i_* and demonstrated that further to the treatment of Jurkat cells with WE, [Ca^2+^]*_i_* significantly increased. The underlying mechanisms need to be explored, however we can hypothesize that the proapoptotic mechanism of WE involves intracellular Ca^2+^ accumulation.

Fruits of *W. somnifera* contain different enzymes including l-asparaginase, which catalyzes the conversion of the aminoacid asparagine to aspartate and ammonia. Through this mechanism, l-asparaginase depletes the cellular levels of asparagine and induces the death of leukemic cells that are unable to synthesize asparagine. l-asparaginase exhibits cytotoxic effects on patient-derived leukemic blasts [29]. A specific phytochemical analysis should be performed to detect the presence of l-asparaginase in our extract. However, in our experimental conditions, the co-treatment of cells with WE plus l-asparagine did not affect the proapoptotic potential of WE. The latter finding may suggest that our extract does not contain l-asparaginase. Thus, it is conceivable that the presence of l-asparaginase does not play a critical role in the cytotoxic activity of WE.

As already mentioned, the main component of our WE is WFA. Many studies have demonstrated the role of WFA inducing oxidative stress as an anticancer strategy on different tumor cell lines, such as prostate cancer, breast cancer, pancreatic cancer, leukemia, and melanoma [30,31,32,33,34]. ROS-mediated apoptosis by WFA was shown to depend on both intrinsic and extrinsic pathways. Mitochondrial membrane potential loss, release of cytochrome c and translocation of Bax, as well as increase in caspase-8 activity were observed together with the decrease of Bid, as crosstalk between intrinsic and extrinsic pathways [32]. Accordingly, we demonstrated that WE increased intracellular ROS levels. The remarkable inhibition of the proapoptotic potential of WE after co-treatment with NAC confirms the key role of ROS production in the apoptosis induced by WE. Similar data were obtained in both estrogen receptor (ER) positive- and ER negative-breast cancer cell lines, where the apoptotic effect of WFA was blunted by the presence of antioxidants [30].

Notably, selectivity by WFA towards cancer cells was observed in pancreatic and breast cancer cells, as compared to normal human fibroblasts and normal human mammary epithelial cell line [30,34]. ROS levels in cancer cells are close to the threshold and a ROS-mediated apoptotic mechanism represents an established indicator of cancer selectivity for an anticancer compound [35].

WE extract caused a dose-dependent ROS generation in Jurkat cells. This finding is in agreement with previous studies reporting the ROS-generating ability of a similar WE extract [32] and some of its components such as WFA [32,33,36,37,38]. ROS generation induced by WE extract reached a plateau after 6 h of incubation. This suggests that the extract rapidly induces a pro-oxidative status in intoxicated cells. Similar results were obtained by Malik [32], who found a significant ROS increase following 1 to 3 h of exposure to WFA [38]. The co-incubation of cells with WE extract and NAC, an established ROS scavenger, quenched the ROS generation induced by WE. This finding is in conformity with previous data that reported that NAC attenuates *Withania*-induced ROS production in several cell lines [32,36,37,38]. Co-incubation with o-Phe, an iron chelator that breaks Fenton reaction and stops ROS generation [39], attenuated ROS generation to a similar extent as NAC. These data strengthen the notion that WE causes the cellular formation of ROS. Since analyses performed on melanoma cell lines treated with WFA recorded mitochondrial ROS generation [33], we investigated whether the mitochondrial respiratory chain is involved in this process. Rot, a prototypical Complex I inhibitor [40], did not affect WE-induced ROS production, suggesting that this complex is not involved in this process and that further studies will be needed to individuate the exact site of ROS production.

ROS production and endoplasmic reticulum (ER) stress are critical event promoting ICD, which is also associated with the expression and/or release of DAMPs [41]. For example, calreticulin is a DAMP usually located on the lumen of ER and translocated on the extracellular membrane in case of ER stress [41]. ER regulates many cellular events including [Ca^2+^]*_i_* levels. Alterations in Ca^2+^ homeostasis causes ER stress [42]. In our experimental settings, WE treatment causes ROS production and increases [Ca^2+^]*_i_* levels. Accordingly, to the best of our knowledge, we demonstrated for the first time the ability of *Withania* to induce ICD starting from the lowest tested concentrations, as indicated by the up-regulation of calreticulin, Hsp-70, Hsp-90, and ATP release. The expression of these molecules increases the immunogenic profile of tumor cells, thus promoting the innate immune system response [43]. However, WFA and WDA that represent the two main constituents of our WE extract did not induce ICD either alone or in association. Recent evidence shows that WFA does not alter the expression of Hsp-90 either in lymphoma or in pancreatic cells, and inhibits Hsp-90 with an ATP-independent mechanism [44,45,46]. The anticancer activity of WFA depends on the inhibition of critical kinases and cell-cycle regulators controlled by Hsp-90 [44,45]. Hsp-90 is a molecular chaperone involved in regulating protein folding and modulating a number of oncogenic client proteins playing a critical role in oncogenesis and cancer progression. Hsp-90 depends upon different co-chaperones for its function. WFA blocks the association of Hsp-90 to Cdc37, *i.e.*, its co-chaperone, thus acting as a potent Hsp-90-client modulating agent [46]. The different activity of the two withanolides and WE could be imputable to the complex nature of WE. In other words, the combined effects of the bioactive molecules of the extract could differently influence DAMPs’ expression.

An immunostimulatory activity of an aqueous/alcoholic (1:1) root extract of *Withania* has been reported on BALB/c mice and on *ex vivo* and *in vitro* macrophages [47]. The immunostimolatory activity of *Withania* together with the induction of ICD represents a promising strategy for the generation of a tumor-specific response.

Finally, we analyzed the genotoxic potential of WE. Our results showed that the treatment of Jurkat cells with WE significantly boosts H2A.X phosphorylation, which is an index of the ability of a compound to interact with DNA thus triggering a genotoxic lesion. However, some recent *in vivo* studies reported the lack of genotoxicity of one of the most important constituents of *Withania*, *i.e.*, WFA, and demonstrated its ability to provide protection against the 7,12-dimethylbenz(a)anthracene-induced genotoxicity [48,49]. As with the different behavior of WE and withanolides in ICD induction, the different genotoxic profile of WE and WFA could be due to the matrix effects. Genotoxic studies on complex products of natural origin are usually performed on single phytochemicals rather than on the product in its complexity. The matrix effect can cause an incomplete release of a key constituent from the vegetal matrix or modulate its bioavailability. This means that the use of toxicity data concerning the pure phytochemical are unsuited for the purposes of assessing the risk derived from the use of the same phytochemical within the complex vegetal matrix [2]. The use of *Withania* is suggested in different contexts including naturopathic care for anxiety [50], anti-infertility care [51], and osteoarthritis care [52]. Its genotoxicity should be carefully considered for an accurate assessment of its risk–benefit profile. It is important to note that the H2A.X phosphorylation test used in our study is able to detect only premutational, thus reparable DNA lesions. For this reason, further experiments are needed to define the net and actual mutagenic effect of the lesions caused by WE and to directly relate the DNA damage to the mutagenic effect.

## 4. Experimental Section

### 4.1. WE Preparation

*Withania somnifera* roots were collected during the balsamic period (summer) and authenticated by Dr. Paolo Scartezzini, Maharishi Ayurveda Product Ltd., Noida, India. The quality control was performed by Vedic Herbs s.r.l. (Caldiero, VR, Italy), which gifted us with a sample of root powder (voucher #12/11). The extract was prepared by mixing 10 g of *Withania* root powder with 100 mL of DMSO. The extract was vortexed for 15 min at room temperature and centrifuged to discard any insoluble part. The experiments and the HPLC analysis were performed using this stock solution of 100 mg/mL.

### 4.2. HPLC Analysis

WE was subjected to RP-HPLC-DAD analysis to identify and quantify the main phytomarkers. The reference compounds WFA, WDA, withanolide B, and withanone were purchased from Extrasynthese, Lyon, France. WFA and WDA were used as external standards to set up and calculate appropriate calibration curves. The analyses were performed using a Jasco modular HPLC (model PU 2089, Jasco Corporation, Tokyo, Japan,) coupled to a diode array apparatus (MD 2010 Plus) linked to an injection valve with a 20 μL sampler loop. The column used was a Kinetex XB-C18 (5 μm, 15 cm × 0.46 cm) with a flow rate of 0.6 mL/min. The analyses were performed at 25 °C with mobile phase and gradient chosen according to literature [17].

Following chromatogram recording, sample peaks were identified by comparing their ultraviolet (UV) spectra and retention time with those of the pure standards. Dedicated Jasco software (PDA version 1.5, Jasco Corporation, 2004) was used to calculate peak area by integration.

### 4.3. Validation

The individual stock solutions of each phytomarkers were prepared in ethanol or acetonitrile. The calibration curves of the considered compounds were prepared within different range: 500–50 μg/mL for WFA, and 100–10 μg/mL for WDA. Each calibration solution was injected into HPLC in triplicate. The calibration graphs were provided by the regression analysis of the peak area of the analytes *versus* the related concentrations. The analysis of the extract was performed under the same experimental conditions. The obtained calibration graphs allowed the determination of the concentration of the phytomarkers inside the extract.

Limit Of Detection (LOD) and LOQ were calculated following the approach based on the standard deviation of the response and the slope for WFA and WDA, on signal and noise ratio for withanolide B and withanone, as presented in the “Note for guidance on validation of analytical procedures: text and methodology”, European Medicine Agency ICH Topic Q2 (R1). The accuracy was reported as percent of recovery and was estimated by adding known amount of analyte in the studied sample.

### 4.4. Cell Cultures

Human T-lymphoblastoid cells (Jurkat) were provided from LGC standards (LGC Group, Middlesex, UK). Cells were grown in suspension in Roswell Park Memorial Institute (RPMI) 1640 supplemented with 10% heat-inactivated bovine serum, 1% penicillin/streptomycin solution, and 1% l-glutamine solution (all obtained from Biochrom, Merck Millipore, Darmstadt, Germany). Cells were incubated at 37 °C with 5% CO_2_. To maintain exponential growth, the cultures were diluted to never exceed the maximum suggested density of 3 × 10^6^ cells/mL.

### 4.5. Cell Treatment

Cells were treated with increasing concentrations of WE (0.0–1.6 mg/mL) for 1, 3, 6 or 24 h, according to the experimental requirements, or with WFA, WDA or WFA plus WDA for 24 h. WFA and WDA were tested at the concentrations found in the WE extract at 0.2, 0.4 and 0.8 mg/mL: 0.23–0.92 μg/mL for WFA; 0.08–0.32 μg/mL for WDA. Etoposide 10 μM and hydrogen peroxide 0.1 mM were used as positive controls. In some experiments, a co-treatment of WE with NAC or l-asparagine was performed.

### 4.6. Analysis of Cell Viability and Induction of Apoptosis

To determine cells’ viability, Guava ViaCount Reagent (Merck Millipore, Darmstadt, Germany) was used according to manufacturer’s instructions. Briefly, cells were appropriately diluted with the reagent containing 7-amino-actinomycin D (7-AAD) and incubated at room temperature in the dark for 5 min before detection with flow cytometer. Furthermore, to discriminate between necrotic and apoptotic events, Guava Nexin Reagent (Merck Millipore) was used. Through the use of 7-AAD and annexin V-phycoerythrin, the assay allows the discrimination of apoptotic and necrotic events. Cells were incubated with the reagent for 20 min at room temperature in the dark and then analyzed via flow cytometry. IC_50_ was calculated by interpolation from dose–response curve. Concentrations ≤ IC_50_ were used in the subsequent experiments.

### 4.7. Cell-Cycle Analysis

After treatment with WE for 24 h, cells were fixed with 70% ice-cold ethanol and, after washing, suspended in 200 μL of Guava Cell Cycle Reagent (Merck Millipore), containing propidium iodide. At the end of incubation at room temperature for 30 min in the dark, samples were analyzed via flow cytometry.

### 4.8. Measurement of [Ca^2+^]_i_

After WE treatment for 6 or 24 h, [Ca^2+^]*_i_* was analyzed by using Fura Red™, AM (Thermo Fisher Scientific, Carlsbad, CA, USA), according to manufacturer’s instructions. Briefly, after treatment, cells were incubated with the dye that freely permeates the cytoplasmic membrane but, once inside the cells, is hydrolyzed by the intracellular esterases and trapped into the cells. The fluorescence of this molecule is enhanced once it binds Ca^2+^. To determine the optimal concentration of dye, a titration of Fura Red^TM^, AM was performed by loading Jurkat cells with a range of concentrations recommended by the manufacturer (1–10 μM). The exposure of cells to Fura Red can cause cell death [53]. Thus, the use of the lowest concentration of Fura Red is recommended. Following this experimental phase, the concentration of 1 μM was adopted.

To detect intracellular calcium levels, Jurkat cells were incubated at 37 °C for 30 min in PBS without calcium and magnesium. This buffer condition allows to detect the intracellular calcium stores and exclude the secondary increase in [Ca^2+^]*_i_* due to Ca^2+^ entry [54]. Moreover, the removal of external Ca^2+^ reduces the non-specific fluctuations in [Ca^2+^]*_i_* normally observed during the first 20–30 s of sample acquisition via flow cytometry. Results are expressed as MFI.

### 4.9. Detection of ROS Production

ROS production was determined after 1, 3, 6, 18 or 24 h of WE treatment by using the probe dihydrorhodamine (DHR, 10 μM) [55], which was added during the last 15 min of incubation. Hydrogen peroxide was used as positive control. Additionally, cells were pre- treated for 30 min with Rot (2 μM) or o-Phe (10 μM) and co-treated for 6 h with WE (0.8 mg/mL). In some experiments, cells were co-treated for 6 h with WE (0.8 mg/mL) plus NAC (10 mM). After three washing in PBS, cellular fluorescence was imaged using a Leica DMLB/DFC300F fluorescence microscope (Leica Microsystems, Wetzlar, Germany) equipped with an Olympus ColorviewIIIu CCD camera (Polyphoto, Milan, Italy). Fluorescence images (100 cells per sample from randomly selected fields) were digitally acquired and processed for fluorescence determination at the single cell level on a personal computer using the public domain program, Image J. Mean fluorescence values were determined by averaging the fluorescence of at least 100 cells/treatment condition/experiment.

### 4.10. Analysis of Calreticulin Translocation, Hsp-70 and Hsp-90 Expression, and ATP Release

After 6 or 24 h of treatment, cells were washed and incubated with phycoerythrin-labeled calreticulin antibody (1:100, Abcam, San Francisco, CA, USA) or isotope-matched negative control (isotypic mouse IgG1 K Alexa Fluor 488^®^) (eBioscience, San Diego, CA, USA).

To analyze Hsp-70 and Hsp-90 expression, cells were incubated with an anti-Hsp-70 or anti-Hsp-90 antibody (1:100, Abcam, for both antibodies) and, after washing, incubated with fluorescein isothyocianate-labeled secondary antibody (1:100, Sigma, Merck Millipore, Darmstadt, Germany) or the isotype control. Mean fluorescence was detected via flow cytometry.

The kit ATPLite™ 1step (Perkin Elmer, Waltham, MA, USA) was used for the detection of ATP extracellular concentration. Jurkat cells were seeded and treated with WE in Hank's Balanced Salt Solution (HBSS) or complete medium for 6 and 24 h, respectively. At the end of incubation, supernatants were collected and treated with 100 μL of ATPLite 1step reagent containing luciferase and D-luciferin. After shaking for 2 min at 700 rpm using the orbital microplate shaker 711/+ (Asal srl, Florence, Italy), luminescence of the samples was measured in a 96-well black plate using the microplate reader Victor X3 (Perkin Elmer).

### 4.11. DNA Damage Analysis

Phosphorylation of histone P-H2A.X was used as marker of WE genotoxic potential. After, 6 h of treatment with WE, cells were fixed, permeabilized and incubated for 30 min in the dark at room temperature with an anti-P-H2A.X-Alexa Fluor^®^ antibody (Merck Millipore). Etoposide 10 μM was used as positive control. Samples were analyzed via flow cytometry.

### 4.12. Flow Cytometry

EasyCyte 5HT (Merck Millipore) was used to perform all flow cytometric analyses, with the exception of the measurements of [Ca^2+^]*_i_* performed by using a FACSCanto II (BD Bioscience, Franklin Lakes, NJ, USA). For each sample, approximately 5000 events were evaluated.

### 4.13. Statistical Analysis

All results are expressed as mean ± SEM of at least three independent experiments. Differences between treatments were assessed by t test or one-way ANOVA and Dunnet or Bonferroni was used as post-tests. All statistical analyses were performed using GraphPad InStat 5.0 version (GraphPad Prism, San Diego, CA, USA, 2007). *p* < 0.05 was considered significant.

## Figures and Tables

**Table 1:** Quantification of withaferin A (WFA) and withanolide A (WDA).

Compound	Amount (μg/mL)	LOD	LOQ	Amount (mg/g of Dried Extract)	Recovery %
WFA	113.65 ± 2.84	6.54 ± 0.11	19.81 ± 0.63	5.68 ± 0.14	96.85 ± 1.98
WDA	39.42 ± 1.44	1.64 ± 0.07	4.96 ± 0.26	1.97 ± 0.07	110.57 ± 2.11
withanolide B	tr	2.03 ± 0.34	6.36 ± 0.65	-	-
withanone	-	1.99 ± 0.29	15.95 ± 1.18	-	-

LOD: limit of detection, LOQ: limit of quantification, tr = trace.
